# Studying the evolutionary potential of ancestral aryl sulfatases in the alkaline phosphatase family with droplet microfluidics

**DOI:** 10.1039/d5an00865d

**Published:** 2026-02-05

**Authors:** Bernard D. G. Eenink, Josephin M. Holstein, Magdalena Heberlein, Carina Dilkaute, Joachim Jose, Florian Hollfelder, Bert van Loo, Erich Bornberg-Bauer, Tomasz S. Kaminski, Andreas Lange

**Affiliations:** a Institute for Evolution and Biodiversity, University of Münster Germany andreas.lange@uni-muenster.de ebb@uni-muenster.de; b Department of Biochemistry, University of Cambridge UK; c Institute of Pharmaceutical and Medicinal Chemistry, University of Münster Germany; d Department of Applied Sciences, Northumbria University Newcastle-upon-Tyne UK; e Department of Protein Evolution, Max Planck Institute for Developmental Biology Tübingen Germany; f Department of Molecular Biology, Institute of Biochemistry, Faculty of Biology, University of Warsaw Warsaw Poland ts.kaminski2@uw.edu.pl

## Abstract

Characterizing the dynamics and functional shifts during protein evolution is essential, both for understanding protein evolution and for rationalizing efficient strategies for *e.g.* enzymes with desired and effective functions. Most proteins organize in families, sets of divergent sequences which share a common ancestor and have a similar structural fold. Here, we study aryl sulfatases, a subfamily of the large and evolutionary old alkaline phosphatase superfamily. We demonstrate how ultrahigh-throughput droplet microfluidics can be used for studying aryl sulfatases and their computationally reconstructed putative common ancestors. We compare the evolvability and robustness of three ancestors and three extant aryl sulfatases which all exhibit catalytic promiscuity towards a range of substrate classes. Using varying mutations rates, eleven libraries were constructed and expressed in single-cell microdroplets. In general, higher mutation rates resulted in wider distribution of active variants but fewer improved variants overall. However, the impact of mutation rate differed between enzymes, with some benefiting from higher and others from lower mutation rate, underscoring the need to test diverse mutagenesis regimes.

## Abstract 2

Extant and reconstructed ancestral aryl sulfatases were compared using directed evolution. Mutant libraries were screened using microfluidic droplet sorting, the activity of each variant was measured and improved variants were recovered.

## Graphical abstract

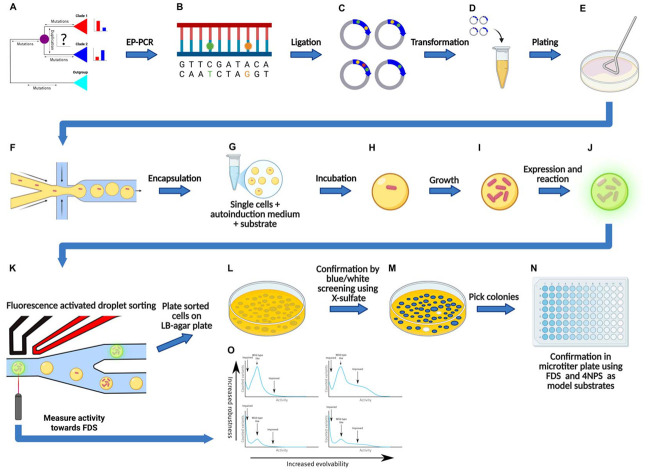

## Introduction

Protein families are believed to have originated from shared ancestors through gene duplication, followed by sequence and functional divergence,^1,2^ while structures generally remain more conserved.^3,4^ Categorizing proteins into families is fundamental to understanding structural and functional connections between proteins.

Enzyme promiscuity is a key factor in the evolution of new functions,^5,6^ providing organisms with moderate activity toward non-standard substrates and lowering the threshold for selective advantage.^6–8^ By turning over non-standard substrates, even before gene duplication,^6^ promiscuous activity can enable the rapid development of biocatalysts for green chemistry and xenobiotic degradation.^9^

Directed evolution mimics natural evolution^10,11^ to improve traits such as catalytic efficiency,^12^ stability,^13^ enantioselectivity,^14^ and medium tolerance.^15^ Success of directed evolution relies on both the kinetic parameters of the starting enzyme but also on the shape of the local fitness landscape.^9,16^ For example, shallow fitness peaks may be easier to reach and variants may be easier maintained due of the sheer number of viable variants in the near vicinity while steeper fitness peaks may be difficult to reach and variants more easily lost (“survival of the flattest”).^17,18^

### Aryl sulfatases

To test the possible effects of the properties of the fitness landscape and the premise that ancestral members of an enzyme family may be either promiscuous or more evolvable or both, enzymes families with many testable variants and accessible substrates are required. One such family are Aryl sulfatases (AS). ASs catalyse the de-esterification of sulfate mono-esters.^19^ Many ASs show catalytic promiscuity towards other substrate classes.^20^ Promiscuous activity can reach high levels, sometimes on par with the catalytic efficiency towards the native substrate,^21,22^ making ASs suitable to investigate the characteristics and evolvability of ancestral enzymes.

**Fig. 1:**
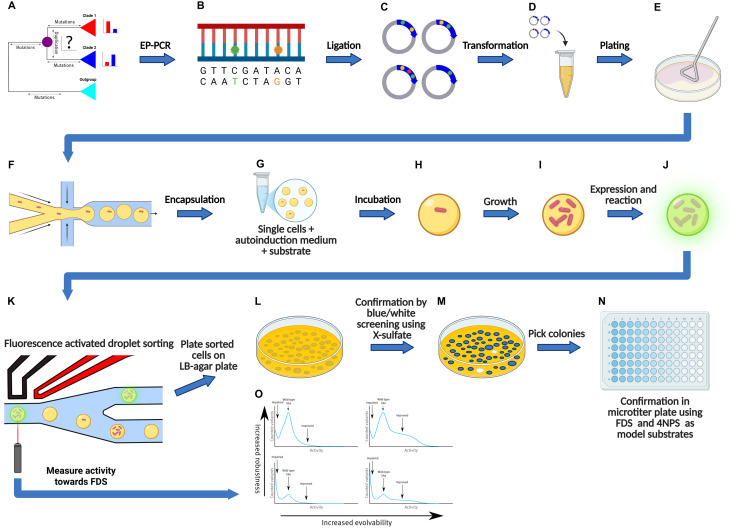
Overview of the protocol used. Ancestral enzymes were reconstructed using ASR (A). epPCR was used to generate mutant libraries of each enzyme (B). Variant libraries were ligated into pBAD-AT-shift in frame with the AIDA autodisplay construct (C). *E. coli* cells were transformed with the libraries (D) and spread on agar plate. Grown colonies were subsequently scraped from agar plate (E) and encapsulated inside a water-in-oil microdroplets together with LB-medium and substrate (F). The water-in-oil droplets were collected in an Eppendorf tube (G) and incubated for growth and expression (H to J). Next, droplets were sorted using fluorescence activated droplet sorting (K). The activity towards FDS in each droplet was measured (O). 0.1% of the most active variants were sorted, plated on LB agar plate (K) and confirmed using blue-white screening with X-sulphate on agar plate and 4NPS and FDS in microtiter plate (N). This figure was prepared with BioRender.com.

Model substrates for ASs are widely available, and can be used to detect sulfate, phosphate and phosphonate esterase activity using a variety of detection methods (Fig. 1).^20^

### Ancestral sequence reconstruction

Ancestral sequence reconstruction (ASR) is used to deduce ancestral sequences at divergent nodes in a phylogenetic tree.^23,24^ Ancestral enzymes exhibit several noteworthy properties, such as high thermostability,^25–27^ catalytic promiscuity,^26,28^ and retaining much higher activity at lower temperatures.^29^ Minor molecular changes can drastically alter enzyme function.^2,30^ Increases in thermostability by 20–30 °C can be achieved.^31–33^ Although debate remains whether ancestral properties reflect a genuine evolutionary trend or an artifact arising from ASR methods,^27,34^ these properties are of interest for directed evolution. Promiscuous enzymes adapt more easily to new functions. Thermostability allows function-enhancing, destabilizing mutations without compromising function.^35^ Studies in ancestral sequence reconstruction generally probe biological rather than intrinsic specificity.^36,37^ Whereas biological specificity is the affinity of a protein for potential targets at varying concentrations, as a result of selection, intrinsic specificity is a general affinity to targets not encountered and thus not the result of selection, that can nonetheless be a basis for novel functionality.^37^

Recently, ASR has been used to create highly stable and functional proteins.^38–42^ Additionally, mutagenesis has been used to further improve proteins derived from ASR, using both rational design,^38^ directed evolution,^39^ or both.^40^ However, previous studies mostly revolve around introduction of mutations in an ancestral backbone, and directed evolution on ancestral enzymes is compared with campaigns previously conducted on extant enzymes, rather than direct comparison in one experimental setup.

In this study, we perform parallel directed evolution campaigns on ancestral and extant enzymes from the AS family using identical parameters, conditions, and experimental setups. This approach enables a direct, high-throughput comparison of the evolutionary trajectories and performance of ancestral *versus* extant enzymes.

### Micro-droplet sorting

Water-in-oil emulsion-based micro-droplet sorting enables the screening of large libraries by encapsulation proteins in picoliter volume droplets.^43–45^ By encapsulating a single cell or plasmid inside each droplet, genotype-phenotype linkage is maintained. Droplet sorting has been used to screen various enzyme classes ultra-high throughput, both *in vivo* and *in vitro*.^46,47^ Although cell lysis is commonly used,^43,45^ by using autodisplay to express enzyme on the cell surface, living cells can be directly recovered.^12,48,49^

**Fig. 2:**
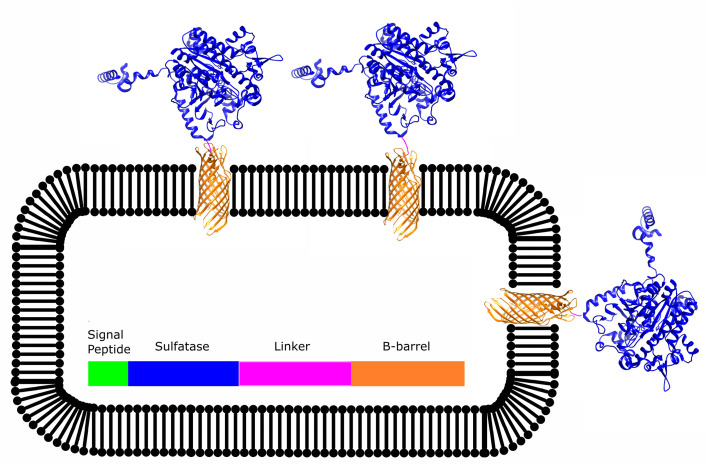
Schematic overview of the sulfatase enzymes displayed on the outer membrane of *E. coli* cells. A signal peptide directs the fused protein towards the periplasmic space where the AIDA β-barrel folds into the outer membrane and displays the passenger enzyme at the surface.^49,51^

Recent studies show inoculating droplets with a single cell followed by cell growth inside droplets can improve sensitivity and recovery while preserving genotype-phenotype linkage.^50,51^ The *E. coli* autodisplay system (Fig. 2)^48,49^ is used to present enzymes, including multimeric enzymes^52,53^ on the cell surface. This technique enables the recovery of live, intact cells after sorting.

### Aims

This study aims to directly compare ancestral and modern enzymes using the same experimental approach. By integrating droplet microfluidics, the *E. coli* autodisplay system (Fig. 2),^48,49^ and ancestral sequence reconstruction (ASR), we analyze ASs and phosphonate monoester hydrolase (PMHs) from the AP superfamily.^20^ This framework enables broader enzyme characterization, with the following objectives: (i) systematically assessing the evolvability (proportion of variants with improved catalytic efficiency over the wild type) and robustness (proportion of variants retaining wild-type-like or better catalytic efficiency) of ancestral proteins, (ii) correlating catalytic efficiency gains observed in high-throughput screens with those in cytosolically expressed, purified proteins, and (iii) comparing the local fitness landscapes of extant and ancestral AS enzymes.

## Materials and methods

### Ancestral reconstruction procedure

The multiple sequence alignment calculation as well as the phylogeny reconstruction were performed based on the amino acid sequences. For the ancestral gene reconstruction, the coding DNA sequences of each protein in the dataset were downloaded from the NCBI RefSeq database^54^ and aligned similarly to the previously calculated protein sequence alignment using the TranslatorX server.^55^ The resulting nucleotide sequence alignment and phylogenetic tree were used as input for the codeml program of the Phylogenetic Analysis by Maximum Likelihood (PAML) software package.^56^ The ancestral nucleotide sequences were reconstructed based on coding DNA sequence alignment. The manual refinement of ancient sequences was based on the maximum parsimony principle, assuming the lowest possible number of changes. Each position of the final alignment was manually inspected in terms of the amino acids presence/absence in the reconstructed ancestor *versus* its descendants. The sequence similarity between the reconstructed and the extant proteins was calculated using blastp, using the presented dataset as a local database. For subsequent cloning experiments, all *Xho*I, *Kpn*I, *Pst*I, *Bam*HI and *Hind*III restriction sites were removed from the reconstructed sequences. The sites were changed by substituting the third base of the codon encompassed in the restriction site to the second most probable base as reconstructed by PAML. At the C-terminus of all sequences, a TAA stop codon was added as the one with highest frequency in *E. coli*. (61% cases according to the GenScript CodonUsageFrequency Table Tool available at https://www.genscript.com/cgibin/tools/codon freq table (accessed 2014/9/15)/64% cases according to the Codon Usage Database.)^57^ To facilitate cloning, linker sequences were added at the N- and C-termini of each reconstructed gene (N-terminus: 5-GTACCCGGGGATCCCTCGAG-3, C-terminus: 5-CTGCAGGGGGACCATGGTCT-3). The sequences were optimized according to the Gen9 synthesis guideline. The *Bsa*I and *Aar*I recognition sites were removed (in the same way as other restriction sites) and the local GC content was adjusted using the Gen9 online optimization tool. The synthetic genes were purchased from Gen9.

### Cloning procedure for cytosol expression

**Fig. 3:**
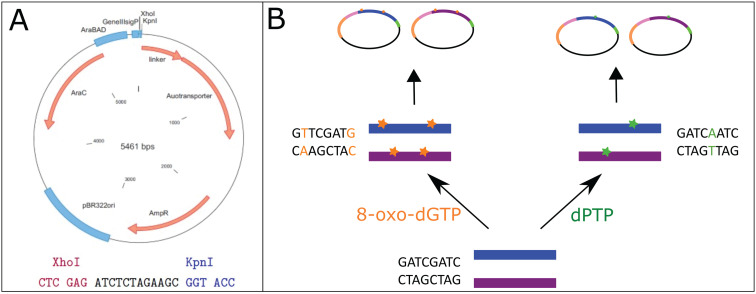
Overview of plasmid engineering used in this study. pBAD-AT-shift (A) was used for all vector engineering. epPCR libraries were generated by spiking the PCR reaction with the synthetic nucleotides 8-oxo-dGTP (yellow) and dPTP (green), which each have a unique substitution profile and a different mutation rate (B).

For the cytosolic expression and purification of enzymes, DNA sequences were cloned into the pASKiba5 + vector (Fig. 3). Ancestral sequences (Anc497, Anc498, Anc499) were cloned using the restriction sites *Pst*I + *Xho*I, while extant sequences (*Sp*AS2, *Sa*AS and *Ak*AS) were cloned using the *Xho*I + *Kpn*I restriction sites. Subsequently, chemically competent *E. coli* TOP10 cells (INOUE method) were transformed with the assembled plasmid.

### Purification of cytosolically expressed enzymes

10 mL of LB-medium containing 50 µL L^−1^ ampicillin was inoculated with *E. coli* cells containing an expression vector for the respective ancestral and extant genes. This pre-culture was grown overnight at 37 °C. 2 L 2× YT medium (16 g L^−1^ tryptone, 10 g L^−1^ yeast extract, 5 g L^−1^ NaCl) was inoculated with the preculture and incubated at 37 °C while shaking until an OD_600_ of between 0.6 and 0.8 was reached. Expression was induced by adding anhydrotetracycline (200 µg L^−1^) followed by overnight growth at 25 °C. Cells were harvested by centrifugation, resuspended in HCl buffer pH 8.0 with EDTA-free Protease Inhibitor Cocktail (Roche). Cells were subsequently lysed by addition of 1× Bugbuster containing Lysonate and incubated for 1 h at room temperature. Cell debris was removed by centrifugation (90 min, 15 000*g*, 4 °C), the supernatant was passed through a 0.45 µm syringe-driven filter. The proteins were purified using affinity chromatography with a Strep-Tactin column (IBA).

Peak fractions were combined, concentrated down to 100 µM. The buffer was exchanged to 50 mM Tris-HCl pH 8.0 by passing the protein solution through PD MiniTrap G-25 columns (GE Healthcare). Protein concentration was determined from absorption at 280 nm. The extinction coefficients and molecular weights (MW) were calculated using ProtParam at ExPASy (https://web.expasy.org/protparam/). Protein aliquots were either used immediately or frozen in liquid nitrogen and stored at −80 °C.

### Reagents

4-NPS substrate was from Thermo-Fisher. FDS substrate was synthesized as previously described.^58^ X-sulfate substrate was from Goldbio.

### Cloning procedure for autodisplay

For the expression of individual variants using autodisplay, DNA sequences were cloned into pBAD-AT-shift vector^59^ (Fig. 3) using the *Xho*I + *Kpn*I restriction sites. Subsequently, chemically competent (INOUE method) *E. coli* E. cloni 10G cells were transformed with the assembled plasmids.

### Library generation methods

Libraries were created using error-prone PCR. In both reactions a mutagenic nucleotide analog, 2′-deoxy-P-nucleoside-5′-triphosphate (dPTP) or 8-oxo-2′-deoxyguanosine5′-triphosphate (8-oxo-dGTP), respectively, was supplemented into a standard Taq DNA polymerase reaction (GoTaq, Promega). These nucleotide analogs can form pairbonds with multiple nucleotides and can be erroneously incorporated during PCR. When these nucleotide analogs get duplicated again, a different nucleotide may be incorporated in their place. Of these synthetic nucleotides, dPTP causes mainly transitions, while 8-oxo-dGTP causes mainly translations.^60^ Subsequently, a recovery PCR, using the same protocol but not incorporating synthetic nucleotides, was performed on the obtained PCR fragments to remove the incorporated nucleotide analogs. Sequences were then cloned into pBAD-AT-shift using the *Xho*I + *Kpn*I restriction sites (Fig. 3). The average mutation rates were verified using Sanger sequencing as in ref. 12.

### Expression of autodisplay constructs

Either glycerol stocks or single colonies on agar plate were used to inoculate 3–10 mL LB medium (100 µg mL^−1^ ampicillin) and incubated overnight at 37 °C. Overnight cultures were used to inoculate fresh LB medium and incubated at 37 °C until an OD_600_ of ±0.5. After reaching OD_600_ cells were transferred to expression temperature and incubated for another hour. Cells were then induced by addition of 0.02% l-arabinose and expressed overnight. After overnight expression, cells were harvested by centrifugation (5 min, 4000*g*), resuspended in 1/3 volume SID buffer (pH 7.2, 7.6 or 8.0), according to previously determined pH optima.^19,61^ Cells were then washed three times by centrifugation (5 min, 4000*g*) and resuspended in a fresh 1× SID buffer solution before measurements.

### Whole cell assays with autodisplayed variants

50–100 µl 1× SID buffer was added to 20–70 µL of resuspended cells for a total volume of 120 µL in a 200 µL microtitre plate. The cell density of each of the cells in each well was measured at OD_600_ before the addition of substrate. 80 µL of 2.5× final concentration (4NP) or (FDS) in 1× SID buffer was added for 200 µL final volume. The reaction is measured by increase of absorption value at (400 nm) (4NP) or fluorescence (*λ*_ex_ = 490 nm, *λ*_em_ = 515 nm).

### Microfluidic droplet screening

Frozen cells were resuspended in autoinduction medium (LB containing ampicillin 100 mg L^−1^), l-arabinose (0.04% (v/v) and 0.2% (v/v) d-glucose). Cell concentration was adjusted to 0.3 cell per droplet volume based on the assumption that OD_600_ = 1 to correspond to 5 × 10^8^*E. coli* cells per mL. Droplets were produced in a double flow-focusing junction chip (design: https://openwetware.org/wiki/DropBase:droplet_generation_2_inlets, design iv: 40 µL). First, the autoinduction medium was mixed 1 : 1 with a solution of 40 µM fluorescein disulfate in SID buffer pH 7.5 just upstream the flow-focusing (FF) droplet generation junction. At the FF junction, fluorinated oil HFE-7500 (3M) containing 1% (v/v) fluorosurfactant-008 (RAN Biotechnologies) was used to break the continuous stream aqueous phase and generate monodisperse 40 pL droplets with an expected cell occupancy of *λ* = 0.35 (assuming Poisson distribution). Flow rates were following: 40 µL min^−1^ for both aqueous phases and 160 µL min^−1^ for the oil/surfactant phase. The droplet formation was monitored on an inverted microscope (SP981, Brunell Microscopes) equipped with a high-speed camera (Miro ex4, Phantom Research). The droplets were collected in a droplet chamber (as described in ref. 62). Generated droplets were incubated for 1 to 3 days at 30 °C.

The droplets were incubated for 3 days in dedicated droplet chambers.^62^ The stability and monodispersity of microfluidic droplets was checked using inverted fluorescence microscopy (EVOS FL, Thermo Fisher) before and after incubation. Additionally, droplet stability was verified during flow and fluorescence detection. Droplets entering the detection channel that deviated from the defined size threshold, due to issues such as unwanted merging during incubation, were not sorted. This additional gating was enabled by automated, high-throughput processing of fluorescence signals using field-programmable gate array (FPGA) electronics and LabVIEW software.

During incubation the cells were aerated^50^ by flushing with fluorinated oil HFE-7500 (3M) containing 1% (v/v) fluorosurfactant-008 at a rate of 4 µL per minute. After 2–3 days the droplets were sorted using FADS as previously described in van Loo *et al.*, 2019.^12^ The LABVIEW script used is available online https://github.com/droplet-lab/spinDrop/tree/main/LabVIEW%20FADS. Droplets were re-injected from the droplet chamber onto a microfluidic chip (design: https://openwetware.org/wiki/DropBase:droplet_electrosorting_3) using HFE7500 (3M) containing 1.5% (v/v) fluorosurfactant-008 (RAN Biotechnologies). Droplets were spaced with oil and pushed through a narrow detection channel to allow single droplet measurement. As the droplets were pushed past a 488 nm laser in the sorting Y-junction, the fluorescent activity inside each droplet was measured by recording emission at 497–553 nm to quantify substrate turnover. When fluorescent activity exceeded a threshold (dependent on wild-type enzyme) pulse and function generators were triggered and generated a square pulse at 8 volts, which was amplified 100-fold by a high-voltage amplifier (610E, Trek) and applied on the sorting device *via* salt-water electrodes (5 M NaCl). This pulse pulled the selected droplets into a collection channel. The threshold was set to 0.1% of total droplets during a calibration and stabilization phase. Droplets sorted during this phase were discarded, once droplet flow was stabilized and threshold established, collection tubes were added to the positive channel channel and waste channel and droplet collection started. Droplets were collected at a high frequency until a total of 1 000 000 droplets were sorted.

A FADS sorting threshold of 0.1% of total droplets was selected. This stringent threshold ensured that only substantially improved variants were sorted and recovered. Considering an average occupancy of 0.35 cells per droplet, this corresponded to sorting approximately the single most active variant per ±300 screened variants. The choice of sorting threshold represents a trade-off between stringency and yield. A lenient threshold risks inclusion of wild-type or wild-type-like variants, thereby increasing the burden of downstream rescreening. Conversely, an overly stringent threshold necessitates screening an excessive number of droplets to recover sufficient positive variants. We believe that a 0.1% threshold represents a suitable compromise under the conditions used here. Future studies may further evaluate how varying this parameter influences the recovery and diversity of improved variants.

The droplets in the positive collection channel were collected in a solution of 100 µL Lucigen recovery medium, 45 µl HFE-7500 (3M) and 5 µL PFO surfactant. The collection tubing was flushed with HFE-7500 (3M) to collect all cells. An additional 500 µL of recovery medium was added after collection and the solution was mixed. The liquid phase was plated on LB + amp plates and incubated overnight.

### Blue-white screening

Cells, recovered from positive droplets after screening, were plated on agar plates (LB + amp) and incubated overnight at 37 °C. Subsequently, cells were scraped from the plate and replated on a fresh LB + amp plate on top of a nitrocellulose filter. After overnight incubation at 37 °C the nitrocellulose filter was transferred to a fresh LB + amp + arabinose plate and incubated overnight at room temperature to induce expression. The next day, the nitrocellulose filters were transferred to a fresh LB + amp + arabinose + X-sulfate plate. Colonies capable of turning over the substrate shifted color to blue. Blue colonies bearing sulfatase activity were counted.

## Results and discussion

### Selection and confirmation of wild-type and ancestral aryl sulfatases

**Fig. 4:**
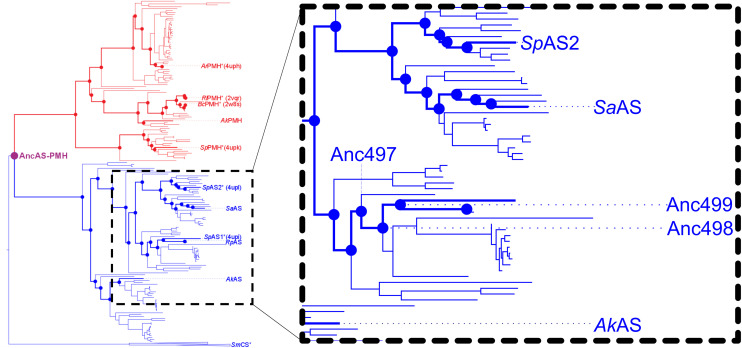
Phylogenetic tree of the AS and PMH families of the AP-superfamily leading back to the common ancestor of AS and PMH. Dots represent inferred ancestral states of the PMHs (red) and ASs (blue). Wildtype enzymes used in the study are labeled. Inferred ancestral enzymes reconstructed and used in the study are labeled in blue. Enzymes with known 3D structures have their PDB code listed after the name.

We reconstructed the aryl sulfatase (AS) and phosphonate monoesterase (PMH) sets of the alkaline phosphatase superfamily to their last common ancestor ASPMH (Fig. 4). We expressed several AS ancestors and characterized these ancestors. We decided to focus on the AS family as ancestral ASs were more amenable to heterologous expression in *E. coli*, as well as a broader availability of model substrates. Thermostability of each wild-type was determined using a thermo-shift assay (Table S2).

To compare the evolvability of extant and ancestral enzymes we selected three ancestral members (Anc497, Anc498 and Anc499, descending from to the presumed common ancestor of ASPMH) and three extant members (*Sp*AS2, *Sa*AS and *Ak*AS) of the AS family of the previously described AP superfamily.^12,20,63^ These extant enzymes were selected to obtain a balanced representation of the AS family, and have previously been successfully expressed and purified^61^ (Fig. 4). Each represents a sub-group of the ASs that descend from ASPMH. We reconstructed the ancestors from *Sp*AS1 towards the root of our set of ASs until the common ancestor of ASs (see Fig. 4). We selected the ancestral enzymes to provide a range of the evolutionary history of ASs.

We successfully expressed each enzyme on the surface of *E. coli*. We confirmed expression and activity of the enzymes by observing the successful turnover of 4-NPS by intact *E. coli* cells surface displaying AS and purification of membrane fractions (Fig. S1). We confirmed turnover of 4-NPS for all ancestral (Anc496, Anc497, Anc498 and Anc499) and extant (*Sp*AS2, *Sa*AS and *Ak*AS) ASs in the surface display assay. Observed enzyme activities were roughly proportional to those observed in cytosolically expressed and purified proteins.

To assay the thermostability of the enzymes, the melting temperature (*T*_m_) of each enzyme was determined using a thermal shift assay (as described in Van Loo *et al.*^20^). The *T*_m_ of the wild-type enzymes selected was between 45.6 °C, and 57.4 °C. All enzymes exhibited *T*_m_ values well above the 30 °C assay temperature, indicating sufficient thermal stability under assay conditions. Among the enzymes tested, Anc497 showed the highest thermostability (57.4 °C), whereas *Ak*AS was the least thermostable (45.6 °C). Anc498, Anc499, *Sp*AS2, and *Sa*AS displayed similar thermostabilities, with *T*_m_ values within 2 °C of one another (Table 1). Initial kinetic parameters varied between enzymes, both ancestral and extant. The *K*_m_ values of the extant enzymes differed by more than an order of magnitude, ranging from 9.9 × 10^−5^ for *Ak*AS to 2.0 × 10^−3^ for *Sp*AS2. The *K*_m_'s of ancestral enzymes were more similar to each other and fell in between the *K*_m_'s of the extant enzymes. The initial *K*_cat_ values of the ancestral enzymes were lower than those of the selected extant enzymes. Moreover, a trend was observed in which deeper ancestral nodes corresponded to lower initial *K*_cat_ values, whereas the extant enzymes displayed slightly higher and more comparable *K*_cat_ values.

**Table 1:** Catalytic parameters of the extant and ancestral wild-types used in this study. Values were measured as described in Van Loo *et al.*^20^

Properties of wild-type and ancestral sulfatases	*K*_cat_	*K*_m_	*K*_cat_/*K*_m_	*T*_m_
Anc497	0.26 ± 0.01	(3.2 ± 0.4) × 10^−4^	(8.1 ± 1.1) × 10^2^	57.4
Anc498	0.56 ± 0.08	(5.6 ± 0.37) × 10^−4^	(1.01 ± 0.14) × 10^3^	49.6
Anc499	1.01 ± 0.4 × 10^1^	(8.4 ± 0.8) × 10^−4^	(1.3 ± 0.1) × 10^4^	48.6
*Sp*AS2	(4.2 ± 0.4) × 10^2^	(2.0 ± 0.3) × 10^−3^	(2.1 ± 0.4) × 10^5^	51.7
*Sa*AS	(2.00 ± 0.04) × 10^2^	(2.8 ± 0.1) × 10^−4^	(7.0 ± 0.4) × 10^5^	49.5
*Ak*AS	(9.7 ± 0.2) × 10^1^	(9.9 ± 0.6) × 10^−5^	(9.9 ± 0.6) × 10^5^	45.6

### Creation and screening of mutant libraries

In order to compare the evolvability of ancestral and extant enzymes, we created error-prone mutant libraries of each ancestral and extant variant. We created mutant libraries through error-prone PCR using either 8-oxo-dGTP or dPTP as mutagenic nucleotides to create mutant libraries with distinct mutational profiles (see Methods: library generation methods). We aimed for a library size of 10^5^ variants to obtain a good coverage of single mutations. We tested each colony by transforming the ligation mixture and counting colonies (Table S1) and the successful insertion of each of the genes was determined by colony PCR (Fig. S2).

After verifying the size and integrity of each library we transformed the remainder of the ligation mix into *E. coli* and divided the cells equally on agar plates. Sulfatase activity was monitored using the FADS with fluorescein disulphate (FDS) as a fluorescent model substrate, in microtiter plates using both FDS and 4-nitrophenol sulfate (4-NPS) as substrates and on agar plates using X-sulfate (5-Bromo-4-chloro-3-indoxyl sulfate), as substrate (Fig. 1).

Ancestral AS sequences were previously inferred towards the root of our set of ASs until the last common ancestor of ASs and PMHs (see Fig. 4) ancestor of AS and PMH. Detailed phylogenetic analysis was performed on extant AS family members.^22^ In this study we describe a parallel directed evolution campaign on three ancestral and three extant AS members for improved catalytic efficiency towards FDS. Autodisplay in *E. coli*, combined with microfluidic FADS, is used to screen and sort living *E. coli* cells presenting unique enzyme variants, with a focus on the shape of the activity distributions of enzyme variants (local fitness landscapes) obtained from FADS sorting (Fig. 1).

All mutant libraries reached the target size of 10^5^ unique variants (Table S1). We determined the amino acid substitution rates for dPTP and 8-oxo-dGTP libraries using Sanger sequencing. In total, we screened 12 libraries, representing two different mutational conditions: (i) dPTP nucleotides (2.3 ± 1.7 mutations) and (ii) 8-oxo-dGTP nucleotides (3.7 ± 2.6 mutations) and six different enzymes, each either inferred ancestral or extant members of AS group of the AP-superfamily. We verified the occupancy of the droplets by observing a sample of droplets using fluoresence microscopy (Fig. S3). For each library we screened 10^6^ occupied droplets (*λ* = 0.35). As a control and to form a baseline of wild-type activity we screened 10^5^ occupied droplets of each respective wild-type enzyme in the same session using the same device for each set. To ensure reproducibility of the microfluidic screening, detector sensitivity was set to comparable levels for each experiment and adjusted when necessary to achieve similar background signal intensities for empty droplets, which accounted for approximately 70% of droplets in each library. When required, measured fluorescence values were additionally normalized to the background signal of empty droplets to enable direct comparison between libraries. Therefore, the reproducibility of these measurements appears to meet the standards of conventional droplet-based microfluidic screening experiments.

With the help of FADS up to 10^6^ variants could be tested for each library, guaranteeing a large coverage of all possible single mutations and full coverage of the targeted 10^5^-member mutant libraries. Usage of the *E. coli* autodisplay system^48,49^ allowed for the direct recovery of recovered intact cells after screening.

All enzymes that had been previously shown to express solubly and active in cytosolic form^12,61^ also expressed well as autodisplay constructs. By using FADS with living cells, it became possible to directly recover the screened cells and plating them on agar instead of recovering and re-transforming the plasmid.

**Fig. 5:**
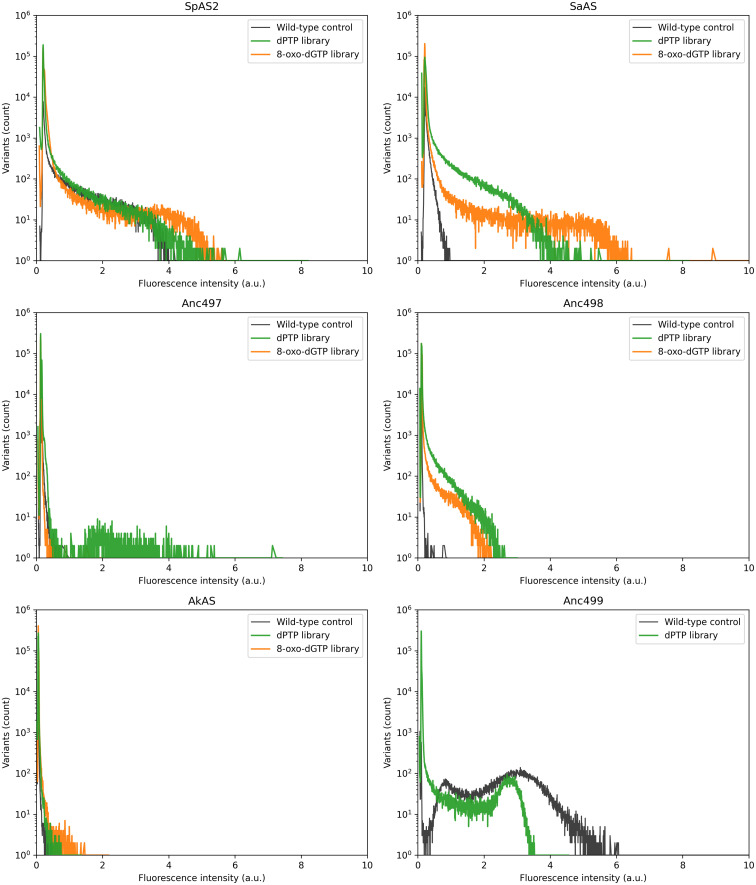
Activity distribution of each ancestral and extant enzyme. 1 000 000 individual droplets were screened using FADS. Libraries shown are dPTP (green) and 8-oxo-dGTP (orange). To measure the baseline activity of each enzyme, 100 000 droplets with wild-type enzymes of each variant were screened (black). Percentage of non-empty droplets exceeding the wild-type average threshold were: Anc497 8-oxo: 0.30%, Anc497 dptp: 11.8%, Anc499 dptp: 0%, *Sp*AS2 oxo: 3.1%, *Sp*AS2 dptp: 3.9%, *Sa*AS oxo: 12.0%, *Sa*AS dptp: 38.7%, *Ak*AS oxo: 56.5%, *Ak*AS dptp: 13.6%. Wild-type threshold values for Anc498 were not informative due to the extreme overlap between empty-droplet and wild-type signal.

While the rates of improvement varied widely (Fig. 5), the overall trend being observed showed a larger increase in improvement in sulfatases having a lower initial activity (Table 1) before introducing mutations. The two libraries with the greatest degree of improvement, Anc498 and *Sa*AS (Fig. 5) came from wild-type enzymes with a *T*_m_ in the middle of the enzymes considered (Table 1). A qualitative effect of mutation rate on enzyme evolvability can also be observed between dPTP (lower mutation rate) and 8-oxo-dGTP (higher mutation rate). Here, we observed that a higher mutation rate leads to a lower improvement in some libraries.

The distribution of extant *Sa*AS broadly followed the theorized pattern, *i.e.* that an increased mutation rate leads to a lower number of improved variants. However, the distribution shows variants with higher activity than in the lower mutation rate library (Fig. 5). This implies that variants with more than one mutation are leading to a maximum improvement, and that the coverage of the library is sufficient to find improved variants with multiple mutations. Even though a majority of variants contain multiple mutants, the library size of 10^5^ can still be assumed to cover the majority of the ±5400 possible single mutants due to oversampling.^12^ Paradoxically, even though most sorted variants contain multiple mutations, the library still does not exhaustively cover all possible (±3 × 10^7^) double mutations. In Anc497 and Anc498 a different trend is found. In the case of Anc497 most improved variants are seen in the dPTP library while for Anc498 most improved variants are seen in the 8-oxo-dGTP library (Fig. 5). This distribution sheds light on the shape of the local fitness landscape, as different mutational rates lead to a quantitatively different outcome. Notably, for Anc499, which had the highest initial catalytic efficiency among the ancestral enzymes, little to no improved variants were observed in the library (Fig. 5). This is reflected in plate re-screening, where no variants with improved activity towards FDS were found (Table 2). Among the other extant enzymes, *Sp*AS2 and *AK*AS we found a shape similar to *Sa*AS although the proportion of improved variants and the highest improvement were much lower, especially in the case of *SpAS2*. This is reflected in the top improvements found for each variant in microtitre plate rescreening (Table 2).

**Table 2:** Top improvements towards FDS and 4-NPS compared to wild type after rescreening in 96-wells plate, including in which library the variant was found

	FDS	4-NPS	Library
Anc497	4.47	4.83	dPTP
Anc498	1.24	4.00	dPTP
Anc499	0.74	1.68	dPTP
*Sp*AS2	52.46	2.07	8-Oxo-dGTP
*Sa*AS	289.17	5.05	dPTP
*Ak*AS	58.04	20.59	8-Oxo-dGTP

### Blue-white and agar plate screening

Blue-white screening of the cells recovered from droplets resulted in an enrichment of blue colonies (Table S3). The blue-white screening step ensured that highly active variants were identified and selected for microtitre plate screening in the next steps.

However, blue-white screening provides only qualitative results and does not yield quantitative information. Therefore, it was used as a pre-screening step prior to microtitre plate screening to ensure that all active variants were selected. Because the wild-type activity levels of some ASs were relatively low, certain variants may appear white due to reduced activity toward X-sulfate, even if they exhibit improved activity toward FDS or 4-NPS. Consequently, blue-white screening was used to identify only positive variants and not negative ones.

Enrichment, defined here as the proportion of recovered cells that show activity towards X-sulfate, varied widely between variants. A trend was observed that dPTP libraries (low mutation rate) consistently showed a higher degree of enrichment than their 8-oxo-dGTP library counterparts (high mutation rate, see Table 2). *Ak*AS was the exception, with a higher enrichment in the 8-oxo-dGTP library, consistent with the relatively poor performance of the *Ak*AS dPTP library in screening (Fig. 5). Furthermore, enrichment was higher in variants with higher initial activity, while several wild types had low activity towards X-sulfate near the detection limit. Hence, the lower enrichment may be caused by catalytic efficiency toward the X-sulfate model substrate not increasing proportionally with the catalytic efficiency towards the FDS substrate, that was used in microfluidic sorting. Thus, white colonies can still contain variants with improved catalytic efficiency toward FDS and 4-NPS.

Subsequently, preliminary microtitre plate screening was carried out on variants picked from the sorted libraries. 176 picked clones of each library were screened. Variants that showed improved catalytic efficiency towards 4-NPS were found in each library, while variants that showed improved catalytic efficiency towards FDS were found in each library except Anc499 (Table 2). The largest improvements were found in variants with low initial activity. When comparing ancestral enzymes to extant enzymes the most notable difference was that while all extant enzymes showed their greatest improvement towards the FDS substrate also used in FADS screening, ancestral enzymes showed their greatest improvements in catalytic efficiency over their wild-types towards the 4-NPS substrate. This difference between substrates is most pronounced in ancestral enzymes Anc498 and Anc499, and reversely extant enzymes *Sp*AS2 and *Sa*AS. The biggest improvements towards both 4-NPS and FDS were observed in ancestral enzymes Anc497 and Anc498 for both FDS and 4-NPS, and extant *Sa*AS for activity towards FDS and *Ak*AS for activity towards 4-NPS, respectively. Coincidentally, these four enzymes showed low initial activity towards FDS. Although only anecdotal, these results hint that variants with lower initial catalytic efficiency may ‘catch up’ and exceed wild-type enzymes with high initial catalytic efficiency even after one round of directed evolution.

### Evolvability of ancestral enzymes

Ancestral enzymes, with their enhanced thermostability and substrate promiscuity, are suggested as promising candidates for directed evolution.^23,35,64^ With the growing accessibility of synthetic DNA, experimental exploration of ancestral enzyme properties has become feasible. To enable direct parallel comparison of ancestral and extant proteins, the mutant libraries were concurrently created using identical methods and backbones. We assessed the evolvability traits of three extant and three ancestral ASs belonging to the AS-PMH branch of the alkaline phosphatase superfamily (Fig. 4). Our aim was to correlate the evolutionary status of the enzyme with its evolvability and robustness.

Using high-throughput fluorescence-activated droplet sorting (FADS), we conducted parallel screening of modern and ancestral enzyme libraries and performed preliminary re-screening of the recovered variants.

The variation in mutation rates highlights the distinct shape of the local fitness landscape for each enzyme. Earlier studies on malate dehydrogenase and lactate dehydrogenase enzymes already showed that a single crucial mutation can switch substrate specificity and greatly impact enzyme activity.^30^ Given that most mutations are either neutral or destabilizing, stabilizing mutations may be necessary to achieve a functional protein with improved or distinct functions. This contrast may explain the disparity between enzyme backbones capable of attaining novel functionality in one step and those needing additional backbone stabilization to achieve novel function.^34,65^

As expected, the observed mutational landscape varied depending on the enzyme and mutational regime. Naturally, increasing the mutation rate would flatten the curve and possibly widen the activity distribution. Given that most mutations are either neutral or detrimental, accumulating multiple mutations increases the likelihood of encountering a deleterious mutation that impairs variant function.

In addition to an increase in deleterious mutations, one would expect an increase in highly improved variants if multiple point mutations are needed to achieve a substantial increase in function. These mutations can either serve as compensatory mutations, necessary to counteract stability loss or folding issues, or as additional mutations directly facilitating changes in function. By introducing multiple mutations in a single round, variants requiring compensatory mutations or epistatic interactions become available. However, each mutation may also cause the enzyme to be non-functional. Here, a balance between the possibility of jumping evolutionary ‘ratchets’ and avoiding the accumulation of deleterious mutations appears. Extra mutations can either be compensatory mutations that offset thermostability loss or folding issues, or all mutations can directly facilitate changes in function. Often, while enzymes with different functions differ in many residues, only a few mutations are responsible and sufficient for a change in function.^30,66^ When comparing this phenomenon, in which a few mutations are able to greatly shift the activity of an enzyme, to our library screening we observed a similar phenomenon especially in the case of *Sa*AS and *Ak*AS (Fig. 5). An interesting case is observed in Anc497, where the dPTP library led to greatly improved variants up to five-fold. Yet, the 8-oxo-dGTP library led to much less improved variants, with the top variant showing only half the improvement of most active dPTP variants, as well as showing significantly fewer improved variants (Fig. 5). These results are consistent with a specific fitness landscape for each variant, such that the optimal number of mutations to achieve an improvement may vary between enzymes. The histograms indicate that ancestral libraries yielded on average fewer improved variants than the extant enzyme *Sa*AS (Fig. 5). However, the ancestors yielded more improved variants than *Sp*AS2 or *Ak*AS. Interestingly, in most ancestral enzymes the lower mutation rate dPTP library yielded more improved variants, while the higher mutation rate 8-oxo-dGTP library yielded more improved variants in extant enzymes.

Furthermore, when improved variants were screened in microtiter plate format, increases in catalytic efficiency towards both FDS as well as 4-NPS (that was not used for microfluidic screening) were found. This effect was most pronounced in ancestral variants Anc497, Anc498 and Anc499, in which the increase catalytic efficiency towards 4-NPS was more substantial than the increase in catalytic efficiency towards FDS (Table 2).

We calculated the percentage of variants exceeding the wild-type (WT) activity threshold (the percentage of non-empty library variants that exceeded the average WT-activity), which ranged from 0% for Anc499 to 38.7% for the *Sa*AS ptp library and 56.5% for the *Ak*AS 8-oxo-dGTP library. Overall, the proportion of variants exceeding WT correlates well with the likelihood of identifying the best-performing variant during microtiter plate screening using the FDS substrate. For Anc499 the top variant identified was below the WT average, whereas for *Sa*AS, the best variant, showing nearly 290-fold enrichment, was also the library with the highest percentage of variants above the WT threshold in droplet-based screening. Together, these results demonstrate quite strong agreement between droplet-based screening and whole-cell assays performed in microtiter plates.

Overall, these results show that ancestral enzymes can show promise as starting points for directed evolution. When it came to extant enzymes, our results showed that the enzymes with greatest starting kinetic parameters showed the least overall improvement, with *Sa*AS outperforming these initially more fit enzymes. Notably, libraries with a lower mutation rate showed a greater proportion of highly improved variants in ancestral libraries, whereas higher mutation rates showed greater proportion of highly improved variants in extant libraries. This characteristic makes ancestral enzymes especially interesting when screening is limited to lower throughputs due to limitations such as substrate or product detection.

## Conclusion

In this study, we integrate microdroplet sorting, autodisplay technology, and ancestral sequence reconstruction (ASR) on the alkaline phosphatase superfamily, focusing on the aryl sulfatases (AS) and phosphonate monoester hydrolases (PMH).^12^ Our objectives were (i) to systematically investigate the evolvability and robustness of ancestral proteins, (ii) to establish a correlation between catalytic efficiency enhancements observed through high-throughput methods and those observed in cytosolically expressed, purified proteins, and (iii) to gain insights into the differences and similarities in the local fitness landscapes of various extant and ancestral AS variants.

We demonstrated that cell-surface-displayed enzyme libraries, grown within droplets, are effective for sorting large libraries—each containing over 10^5^ variants—in parallel within a practical time-frame, as well as applying droplet microfluidics to screen proteins derived from ancestral sequence reconstruction for the first time.

We conclude that, in addition to screening of directed evolution libraries, microfluidic FADS sorting and recovery of autodisplayed proteins can be utilized for a variety of applications in high-throughput screening and sorting of proteins. An example would be the screening of synthetic libraries of proteins derived from multiplexed gene synthesis.^67,68^

When comparing the variant activity histograms of the tested Arylsulfatases (ASs), we found that evolvability was maximized under a lower mutation rate for ancestral enzymes while the extant enzymes benefited from a higher mutation rate. On the other hand, for the ancestral libraries the lower mutation rate dPTP library resulted in a greater proportion of highly improved variants (Fig. 5), demonstrating a qualitative difference in the ideal mutation rate of different enzymes.

The interpretation of data from histograms and microtiter plate rescreens points towards qualitative differences in ideal mutation rate, in which the ancestral enzymes studied show a higher rate of improvement at a lower mutation rate. This work lacks detailed kinetic and structural analysis of improved variants. Further work is required to elucidate the kinetic parameters and structural properties of the improved variants and solidify the conclusions found.

In the future, this study could be followed up with a much more thorough biochemical characterization of improved variants, including detailed kinetics of purified enzymes and elucidation of crystal structures, as well as introduction of the mutations found in the screening into corresponding positions in different enzyme backbones.

Reconstructed ASs showed a propensity towards requiring fewer mutations for improved function. Therefore, ancestral enzymes, particularly in the context of ASs, can serve as valuable initial scaffolds for rapidly achieving significant enhancements in enzyme activity.

Additionally, “imperfect reconstruction”—stemming from less than 100% certainty in assigning residues at ambiguous sites^69^—can be refined through high-throughput directed evolution. Comprehensive coverage of single mutations includes all one-amino acid-off alternative reconstructions, allowing for the selection of variants that enhance functionality.

An initial directed evolution campaign with multiple (both ancestral and extant) enzymes increases the odds of—success, as the candidate with optimal initial catalytic efficiency may not have a traversable, single/double mutation route towards desired function.

Accuracy *versus* throughput is a consideration throughout the experiment. This leads to a funnel-like progress where high-throughput screening is used to select improved variants from a large pool, which are then further characterized using lower throughput microtitre plate screening of variants and finally purified protein.

As ASs typically show promiscuity towards other substrate classes the concept of a low initial activity construct with a broad fitness landscape can be extended towards different enzymes with a promiscuous activity towards the desired reaction.

It should be cautioned that screening towards a limited number of substrates only investigates biological specificity and not intrinsic specificity.^37^ As of such, while ancestral enzymes may be more biologically generalist, a trend linking between ancestrally reconstructed sequences and reduced intrinsic specificity has not been shown, and might not be expected.^37^

These results also open up several lines of inquiry for future work. A first line of inquiry is a longer directed evolution campaign starting from improved variants of the best performing ancestral and extant enzyme. Another avenue is expanding the screening from sulfate esters (4-NPS and FDS) towards substrates for promiscuous activity towards phosphonate esters and phosphate mono- and di-esters. A further path is screening in parallel through a larger array of conditions such as higher mutation rates or differently biased libraries.

## Author contributions

Overall study was conceptualised by B. D. G. E, F. H. B. V., E. B. and J. J. The experimental design was largely performed by B. D. G. E., T. S. K., E. B., J. J., F. H., and B. V; B. D. G. E., M. H., J. M. H., A. L., C. D., T. S. K. and B. V. performed experiments; B. D. G. E., T. S. K.; A. L. and E. B. drafted the first major version of the manuscript with improvements and directions from all other authors.

## Conflicts of interest

The authors declare that they have no known competing financial interests or personal relationships that could have appeared to influence the work reported in this paper.

## Supplementary Material

AN-151-D5AN00865D-s001

## Data Availability

Data for this article, including FADS screening data and the DNA sequences of the proteins used are available at Zenodo at https://doi.org/10.5281/zenodo.18506510. Supplementary information (SI) is available. See DOI: https://doi.org/10.1039/d5an00865d.

## References

[cit1] Ohno S. , in Evolution by gene duplication, Springer Berlin, Heidelberg, 1970

[cit2] Voordeckers K. (2012). *et al.*, Reconstruction of Ancestral Metabolic Enzymes Reveals Molecular Mechanisms Underlying Evolutionary Innovation through Gene Duplication. PLoS Biol..

[cit3] Murzin A. G., Lesk A. M., Chothia C. (1994). Principles determining the structure of β-sheet barrels in proteins II. The observed structures. J. Mol. Biol..

[cit4] Ingles-Prieto A. (2013). *et al.*, Conservation of protein structure over four billion years. Structure.

[cit5] Gupta R. D. (2016). Recent advances in enzyme promiscuity. Sustainable Chem. Processes.

[cit6] Copley S. D. (2017). Shining a light on enzyme promiscuity. Curr. Opin. Struct. Biol..

[cit7] O'Brien P. J., Herschlag D. (2001). Functional interrelationships in the alkaline phosphatase superfamily: Phosphodiesterase activity of E-coli alkaline phosphatase. Biochemistry.

[cit8] Pandya C., Farelli J. D., Dunaway-Mariano D., Allen K. N. (2014). Enzyme Promiscuity: Engine of Evolutionary Innovation. J. Biol. Chem..

[cit9] Yang G. (2019). *et al.*, Higher-order epistasis shapes the fitness landscape of a xenobioticdegrading enzyme. Nat. Chem. Biol..

[cit10] Arnold F. H. (1998). Design by Directed Evolution Some Directed Evolution Experiments. Acc. Chem. Res..

[cit11] Romero P. A., Arnold F. H. (2009). Exploring protein fitness landscapes by directed evolution. Nat. Rev. Mol. Cell Biol..

[cit12] Van Loo B. (2019). *et al.*, High-Throughput, Lysis-free Screening for Sulfatase Activity Using *Escherichia coli* Autodisplay in Microdroplets. ACS Synth. Biol..

[cit13] Giver L., Gershenson A., Freskgard P. O., Arnold F. H. (1998). Directed evolution of a thermostable esterase. Proc. Natl. Acad. Sci. U. S. A..

[cit14] Van Loo B. (2004). *et al.*, Directed Evolution of Epoxide Hydrolase from A. radiobacter toward Higher Enantioselectivity by Error-Prone PCR and DNA Shuffling. Chem. Biol..

[cit15] Mate D. (2013). *et al.*, Blood Tolerant Laccase by Directed Evolution. Chem. Biol..

[cit16] Zheng J., Payne J. L., Wagner A. (2019). Cryptic genetic variation accelerates evolution by opening access to diverse adaptive peaks. Science.

[cit17] Bornberg-Bauer E., Chan H. S. (1999). Modeling evolutionary landscapes: Mutational stability, topology, and superfunnels in sequence space. Proc. Natl. Acad. Sci. U. S. A..

[cit18] Wilke C. O., Wang J. L., Ofria C., Lenski R. E., Adami C. (2001). Evolution of digital organisms at high mutation rates leads to survival of the flattest. Nature.

[cit19] Van Loo B. (2010). *et al.*, An efficient, multiply promiscuous hydrolase in the alkaline phosphatase superfamily. Proc. Natl. Acad. Sci. U. S. A..

[cit20] Van Loo B. (2019). *et al.*, Balancing Specificity and Promiscuity in Enzyme Evolution: Multidimensional Activity Transitions in the Alkaline Phosphatase Superfamily. J. Am. Chem. Soc..

[cit21] Pabis A., Duarte F., Kamerlin S. C. L. (2016). Promiscuity in the Enzymatic Catalysis of Phosphate and Sulfate Transfer. Biochemistry.

[cit22] Van Loo B. (2018). *et al.*, Structural and Mechanistic Analysis of the Choline Sulfatase from Sinorhizobium melliloti: A Class I Sulfatase Specific for an Alkyl Sulfate Ester. J. Mol. Biol..

[cit23] Gumulya Y., Gillam E. M. J. (2017). Exploring the past and the future of protein evolution with ancestral sequence reconstruction: the ‘retro approach to protein engineering. Biochem. J..

[cit24] Merkl R., Sterner R. (2016). Ancestral protein reconstruction: Techniques and applications. Biol. Chem..

[cit25] Romero-Romero M. L., Risso V. A., Martinez-Rodriguez S., Ibarra-Molero B., Sanchez-Ruiz J. M. (2016). Engineering ancestral protein hyperstability. Biochem. J..

[cit26] Risso V. A., Gavira J. A., Mejia-Carmona D. F., Gaucher E. A., Sanchez-Ruiz J. M. (2013). Hyperstability and substrate promiscuity in laboratory resurrections of precambrian B-lactamases. J. Am. Chem. Soc..

[cit27] Wheeler L. C., Lim S. A., Marqusee S., Harms M. J. (2016). The thermostability and specificity of ancient proteins. Curr. Opin. Struct. Biol..

[cit28] Babkova P., Sebestova E., Brezovsky J., Chaloupkova R., Damborsky J. (2017). Ancestral haloalkane dehalogenases show robustness and unique substrate specificity. ChemBioChem.

[cit29] Furukawa R., Toma W., Yamazaki K., Akanuma S. (2020). Ancestral sequence reconstruction produces thermally stable enzymes with mesophilic enzyme-like catalytic properties. Sci. Rep..

[cit30] Boucher J. I. , Jacobowitz J. R., Beckett B. C., Classen S. and Theobald D. L., An atomicresolution view of neofunctionalization in the evolution of apicomplexan lactate dehydrogenases, in eLife, ed. M. Levitt, 2014, vol. 3, p. e02304 10.7554/eLife.02304 PMC4109310 24966208

[cit31] Harris K. L. (2022). *et al.*, Ancestral Sequence Reconstruction of a Cytochrome P450 Family Involved in Chemical Defense Reveals the Functional Evolution of a Promiscuous, Xenobiotic-Metabolizing Enzyme in Vertebrates. Mol. Biol. Evol..

[cit32] Rozi M. F. A. M., Rahman R. N. Z. R. A., Leow A. T. C., Ali M. S. M. (2022). Ancestral sequence reconstruction of ancient lipase from family I.3 bacterial lipolytic enzymes. Mol. Phylogenet. Evol..

[cit33] Joho Y. (2023). *et al.*, Ancestral Sequence Reconstruction Identifies Structural Changes Underlying the Evolution of Ideonella sakaiensis PETase and Variants with Improved Stability and Activity. Biochemistry.

[cit34] Trudeau D. L., Kaltenbach M., Tawfik D. S. (2016). On the Potential Origins of the High Stability of Reconstructed Ancestral Proteins. Mol. Biol. Evol..

[cit35] Alcalde M. (2016). When directed evolution met ancestral enzyme resurrection. Microb. Biotechnol..

[cit36] Wheeler L. C., Anderson J. A., Morrison A. J., Wong C. E., Harms M. J. (2018). Conservation of Specificity in Two Low-Specificity Proteins. Biochemistry.

[cit37] Wheeler L. C., Harms M. J. (2021). Were Ancestral Proteins Less Specific?. Mol. Biol. Evol..

[cit38] Gumulya Y. (2018). *et al.*, Engineering highly functional thermostable proteins using ancestral sequence reconstruction. Nat. Catal..

[cit39] Gomez-Fernandez B. J., Risso V. A., Rueda A., Sanchez-Ruiz J. M., Alcalde M. (2020). Ancestral resurrection and directed evolution of fungal mesozoic laccases. Appl. Environ. Microbiol..

[cit40] Keser M. (2025). *et al.*, Stable and Promiscuous Galactose Oxidases Engineered by Directed Evolution, Atomistic Design, and Ancestral Sequence Reconstruction. ACS Synth. Biol..

[cit41] Thomas A., Cutlan R., Finnigan W., van der Giezen M., Harmer N. (2019). Highly thermostable carboxylic acid reductases generated by ancestral sequence reconstruction. Commun. Biol..

[cit42] Chiang C. H., Wang Y., Hussain A., Brooks C. L., Narayan A. R. (2024). Ancestral Sequence Reconstruction to Enable Biocatalytic Synthesis of Azaphilones. J. Am. Chem. Soc..

[cit43] Mair P., Gielen F., Hollfelder F. (2017). Exploring sequence space in search of functional enzymes using microfluidic droplets. Curr. Opin. Chem. Biol..

[cit44] Levin I., Aharoni A. (2012). Evolution in microfluidic droplet. Chem. Biol..

[cit45] Gielen F. (2016). *et al.*, Ultrahigh-throughput-directed enzyme evolution by absorbance activated droplet sorting (AADS). Proc. Natl. Acad. Sci. U. S. A..

[cit46] Yang J., Tu R., Yuan H., Wang Q., Zhu L. (2021). Recent advances in droplet microfluidics for enzyme and cell factory engineering. Crit. Rev. Biotechnol..

[cit47] Gantz M., Neun S., Medcalf E. J., van Vliet L. D., Hollfelder F. (2023). UltrahighThroughput Enzyme Engineering and Discovery in In Vitro Compartments. Chem. Rev..

[cit48] Jose J. (2006). Autodisplay: Efficient bacterial surface display of recombinant proteins. Appl. Microbiol. Biotechnol..

[cit49] Jose J., Maas R. M., Teese M. G. (2012). Autodisplay of enzymes-Molecular basis and perspectives. J. Biotechnol..

[cit50] Zurek P. J., Hours R., Schell U., Pushpanath A., Hollfelder F. (2021). Growth amplification in ultrahigh-throughput microdroplet screening increases sensitivity of clonal enzyme assays and minimizes phenotypic variation. Lab Chip.

[cit51] Eenink B. D. G. (2022). *et al.*, Vector redesign and in-droplet cell-growth improves enrichment and recovery in live Escherichia coli. Microb. Biotechnol..

[cit52] Lenz F., Zurek P., Umlauf M., Tozakidis I. E., Jose J. (2020). Tailor-made β-glucosidase with increased activity at lower temperature without loss of stability and glucose tolerance. Green Chem..

[cit53] Gesing K. (2024). *et al.*, Surface Display and Engineering of Laccase CotA for Increased Growth of Pseudomonas putida on Lignin. ChemCatChem.

[cit54] Tatusova T., Ciufo S., Fedorov B., O’Neill K., Tolstoy I. (2014). RefSeq microbial genomes database: New representation and annotation strategy. Nucleic Acids Res..

[cit55] Abascal F., Zardoya R., Telford M. J. (2010). TranslatorX: Multiple alignment of nucleotide sequences guided by amino acid translations. Nucleic Acids Res..

[cit56] Yang Z. (2007). PAML 4: Phylogenetic analysis by maximum likelihood. Mol. Biol. Evol..

[cit57] Nakamura Y., Gojobori T., Ikemura T. (2000). Codon usage tabulated from international DNA sequence databases: Status for the year 2000. Nucleic Acids Res..

[cit58] Scheigetz J., Gilbert M., Zamboni R. (1997). Synthesis of fluorescein phosphates and sulfates. Org. Prep. Proced. Int..

[cit59] Schüürmann J., Quehl P., Lindhorst F., Lang K., Jose J. (2017). Autodisplay of glucose-6phosphate dehydrogenase for redox cofactor regeneration at the cell surface. Biotechnol. Bioeng..

[cit60] Zaccolo M., Williams D. M., Brown D. M., Gherardi E. (1996). An approach to random mutagenesis of DNA using mixtures of triphosphate derivatives of nucleoside analogues. J. Mol. Biol..

[cit61] Heberlein M. , Evolution of substrate specificity in the alkaline phosphatase superfamily, University of Münster, PhD thesis, 2016

[cit62] Neun S. , Zurek P. J., Kaminski T. S. and Hollfelder F., in Methods in Enzymology, ed. D. S. Tawfik, 2020, pp. 317–343 10.1016/bs.mie.2020.06.002 32896286

[cit63] Buchholz P. C., Van Loo B., Eenink B. D., Bornberg-Bauer E., Pleiss J. (2021). Ancestral sequences of a large promiscuous enzyme family correspond to bridges in sequence space in a network representation. J. R. Soc., Interface.

[cit64] Trudeau D. L., Tawfik D. S. (2019). Protein engineers turned evolutionists—the quest for the optimal starting point. Curr. Opin. Biotechnol..

[cit65] Gupta R. D., Tawfik D. S. (2008). Directed enzyme evolution via small and effective neutral drift libraries. Nat. Methods.

[cit66] Pillai A. S., Hochberg G. K., Thornton J. W. (2022). Simple mechanisms for the evolution of protein complexity. Protein Sci..

[cit67] Plesa C., Sidore A. M., Lubock N. B., Zhang D., Kosuri S. (2018). Multiplexed gene synthesis in emulsions for exploring protein functional landscapes. Science.

[cit68] Sidore A. M., Plesa C., Samson J. A., Lubock N. B., Kosuri S. (2020). DropSynth 2.0: Highfidelity multiplexed gene synthesis in emulsions. Nucleic Acids Res..

[cit69] Eick G. N., Bridgham J. T., Anderson D. P., Harms M. J., Thornton J. W. (2016). Robustness of reconstructed ancestral protein functions to statistical uncertainty. Mol. Biol. Evol..

